# Digital Health Apps and Web-Based Platforms to Support the Prevention and Management of Snakebite Envenoming: Scoping Review

**DOI:** 10.2196/83744

**Published:** 2026-06-02

**Authors:** Deborah Hosemann, Oliver Gries, Jade Dean Rae, Thao Vi Tran, Philipp Sprengholz, Lars Korn, Thi Thien Thanh Pham, Thi Anh Thu Dang, Benno Kreuels

**Affiliations:** 1 Research Group Neglected Diseases and Envenoming, Bernhard Nocht Institute for Tropical Medicine, Bernhard-Nocht-Straße 74, Hamburg, 20359, Germany, 49 40285380 ext 723; 2 Faculty of Public Health, University of Medicine and Pharmacy, Hue, Vietnam; 3 Institute of Psychology, University of Bamberg, Bamberg, Germany; 4 Research Group Health Communication, Bernhard Nocht Institute for Tropical Medicine, Hamburg, Germany; 5 Institute for Planetary Health Behaviour, University of Erfurt, Erfurt, Germany

**Keywords:** snakebite, neglected tropical diseases, digital health, mobile health, mHealth, digital health application, artificial intelligence, AI

## Abstract

**Background:**

Neglected tropical diseases disproportionately affect underserved populations, with snakebite envenoming (SBE) remaining one of the most overlooked, despite its significant global burden. Digital health applications (DHAs) offer potential to improve prevention, care, and resource management, especially when integrated into digital health interventions. However, despite growing interest, evidence and structured evaluations are limited, making it difficult to assess their impact without a clear overview of existing tools.

**Objective:**

This scoping review aims to provide the first comprehensive mapping of DHAs for SBE, highlighting their potential to strengthen the World Health Organization (WHO) strategy while underscoring the urgent need for structured evaluation, improved quality, and strategic integration to enhance prevention, treatment, and coordination efforts.

**Methods:**

This review followed the Joanna Briggs Institute and PRISMA-ScR (Preferred Reporting Items for Systematic Reviews and Meta-Analyses Extension for Scoping Reviews) guidelines, with a protocol registered on the Open Science Framework. We searched the PubMed database, app stores, and Google for DHAs between September 24 and 26, 2024, addressing snakebite prevention or treatment. To be included, the DHA had to be accessible via the recorded link, contain a description with snakebite-related features (eg, identification, first aid, and treatment), and allow user interaction. Descriptions had to appear in abstracts, app store listings, or website text. Results were grouped by type (mobile- or web-based) and by WHO region. Furthermore, we examined the 2 most common features: first aid and snake identification. First aid content was benchmarked against global guidelines, while identification methods were categorized, and selected artificial intelligence (AI)–based identification apps were exploratively tested using images of medically significant snakes.

**Results:**

A total of 52 eligible results were included, of which 94.2% (49/52) were mobile apps and 5.8% (3/52) were web-based. Regional focus varied, with most apps targeting South-East Asia (n=11, 21.2%), the Americas (n=9, 17.3%), and the Western Pacific (n=5, 9.6%). However, these numbers largely reflect concentration in just a few countries, namely India (n=10, 19.2%), the United States (n=5, 9.6%), and Australia (n=5, 9.6%). The most frequent feature was snake identification support, for example, through photo upload and algorithm-based recognition. However, AI-driven identification often lacked clarity and performed inconsistently in testing. First aid guidance was also common, but only a handful of apps offered comprehensive, evidence-based advice, while others omitted key steps or recommended unsafe practices.

**Conclusions:**

This review provides the first structured evaluation of DHAs for SBE and offers a reproducible framework for assessing digital solutions across neglected tropical diseases. By highlighting key gaps and proposing a foundation for integration into national strategies, it supports the development of equitable, evidence-based digital health innovation in underserved areas.

## Introduction

Snakebite envenoming (SBE) is currently classified by the World Health Organization (WHO) as one of the 21 neglected tropical diseases (NTDs). Even within the NTD portfolio, SBE receives limited attention and resources, despite global estimates of 2.7 million SBE cases annually and an estimated 81,000 to 138,000 deaths each year [1-3]. The WHO’s snakebite prevention and control strategy aims to halve snakebite deaths and morbidity by 2030, by focusing on four priority areas: (1) empowering and engaging communities; (2) ensuring safe and effective treatment; (3) strengthening health systems; and (4) increasing partnerships, coordination, and resources [1]. Progress has been made since this goal was set in 2019, but it remains slow [4].

Digital health interventions (DHIs), which are specifically designed to address health functions through digital means, can enhance access to treatment, health care provider training, collaboration, and resource distribution [5]. DHIs often use digital health applications (DHAs), such as telemedicine, mobile apps, or web-based platforms [6]. Despite the recognized value of DHIs in advancing universal health coverage, evidence of successful implementation for NTDs, including SBE, remains limited [7]. One of the few well-documented examples is in skin NTDs, where mHealth apps, teledermatology, and artificial intelligence (AI)–driven detection tools have improved diagnosis and management in resource-limited settings [7-9]. Additionally, the WHO’s “Skin NTDs app” demonstrates how targeted mHealth apps can support health care workers in making informed clinical decisions, which improve patient care [10].

In SBE, DHIs have the potential to strengthen all 4 WHO priority areas. First, they could empower communities by providing easily accessible first aid and prevention advice. Second, an easy-to-access digital guide outlining safe and effective treatment protocols can enhance the knowledge and capabilities of health care workers. Third, digital reporting applications could be used to collect data on cases that present to the health facilities, helping to fill existing data gaps. Fourth, DHIs could facilitate collaboration among hospitals, nongovernmental organizations, researchers, and patients, improving coordination, treatment, and data sharing. These advancements could streamline prevention, care, and resource management for better outcomes. Despite the rise of DHAs for SBE, there is a lack of evidence, integration within broader DHIs, and structured evaluations. In the absence of a clear overview of existing applications, assessing their impact remains a challenge. Therefore, a comprehensive review of the current landscape for SBE is essential to inform and guide future efforts.

This scoping review systematically identifies currently available DHAs for SBE management, maps their distribution by WHO region, and summarizes their functions. With this review, we aim to provide an overview of the existing DHAs for SBE, laying the foundation to reduce redundancy, foster collaboration, optimize priorities, and improve resource allocation in line with the WHO’s goals.

## Methods

This review was structured according to the Joanna Briggs Institute’s *Manual for Evidence Synthesis on Scoping Reviews*, the PRISMA-ScR (Preferred Reporting Items for Systematic Reviews and Meta-Analyses Extension for Scoping Reviews) checklist (Checklist 1), and design guidelines for scoping reviews, as outlined in the protocol registered with the Open Science Framework on August 2, 2024 [11-13].

### Search Strategy

We searched PubMed database for literature on DHAs for SBE, as well as the Google Play Store, Apple App Store (using fnd.io [14]), and Google search engine for digital applications related to SBE. Search terms for PubMed database were (“snakebite” OR “snake bite”) AND (“digital health” OR “telemedicine” OR “telehealth” OR “mHealth” OR “m-Health” OR “eHealth” OR “E-Health” OR “health app” OR “digital health intervention” OR “digital health technology” OR “healthcare app” OR “mobile health app” OR “remote healthcare” OR “digital diagnosis” OR “digital treatment” OR “digital tool” OR “digital surveillance” OR “digital monitoring” OR “AI”). As Boolean searching did not work in app stores, these were searched by each search term individually (“snakebite,” “snake bite,” “snake identification,” “snake surveillance,” “snakebite surveillance,” “snakebite diagnosis,” “snakebite treatment,” “snakebite first aid,” “snakebite education,” “snakebite prevention”). The internet search was kept broad to try to capture as much as possible (“app” OR “digital health” OR “eHealth” OR “mHealth” AND “snakebite” OR “snake bite”). The complete search strategies and translations are detailed in Multimedia Appendix 1 (Search Queries). All searches were first conducted in English. The Google search was repeated in 13 languages. The first 50 nonsponsored results were selected from each Google search. Non-English results were translated using Google Translate or DeepL [15,16]. Applications known to the author team that were not retrieved through the user-centric search were subsequently added as expert knowledge and screened against the same eligibility criteria.

The searches were performed between September 24 and 26, 2024, from a German IP address. Further details are provided in the published protocol [13].

### Selection Criteria

To be eligible for inclusion, the application had to be accessible through the recorded link and include a description that included features relevant to the treatment or prevention of snakebites in humans (eg, snake identification, snake information, first aid, and treatments). These descriptions had to be available in the abstract (for published literature), app store description or images (Google Play Store and Apple App Store), or website text (Google search). Users had to be able to perform actions in the application through buttons, forms, or menus.

### Study Selection

All search results were exported to a data extraction template in Microsoft Excel (Multimedia Appendix 2) with the following information: publisher or source name, app title (app name, website, or article title), link to the result, search type, and language. After removing duplicates within the searches and results with invalid links, the remaining results were screened for inclusion according to the selection criteria. If an application appeared in multiple searches, it was included only once. When identified in both an app store and through a Google search, the app store version was included in the results. If an app was available in both the Apple App Store and Google Play Store, only the latter was included. Excluded results were documented with exclusion reasons. Two reviewers conducted screening and extraction in duplicate.

### Data Extraction

For the included applications, we extracted the data policy availability, associated access costs, features offered (grouped according to those with a human focus [prevention, first aid, and advanced treatment], snake focus [snake identification and habitat information], or other focus [eg, snake rescue]), the region and/or country if applicable, the platform type (mobile- or web-based), and whether the application was snake-specific or not. In addition to the data included in the original study protocol, we decided during the search to further record user ratings (count and stars), downloads (available for Google Play Store), app store category, device availability (iOS/Android), release date, and the last update before November 18, 2024, if available.

Data were extracted iteratively from online sources for more than 2 months. This resulted in missing values for some of the apps that became unavailable later in the extraction period. While all core variables, such as features and region, had already been recorded before the apps became inaccessible, assessments of AI and first aid information could not be performed for all apps.

Discrepancies were resolved through discussion and cross-verification. Data extraction was completed by November 19, 2024. Any deviations from the original protocol, along with their rationale, are detailed in Multimedia Appendix 3.

### Ethical Considerations

This study synthesized information from publicly accessible sources (including app store listings and publicly available websites) and did not involve human participants, interventions, or individual-level identifiable data. Therefore, ethics approval, informed consent, and participant compensation were not applicable.

### Synthesis of Results and Content Evaluation

Because of regional download restrictions and the lack of a standardized evaluation framework, we were unable to assess all app features systematically. Instead, we focused on the 2 most common features: first aid and snake identification.

First aid content was compared with globally applicable guidelines from Health Action International and the Global Snakebite Initiative [17]. The results can be found in Multimedia Appendix 4. For snake identification features, we classified apps into different categories based on the methodology provided by the app provider. In cases where apps reported AI-based identification features, we reviewed publisher descriptions and user feedback.

We then conducted exploratory testing of 3 different AI-based identification apps to assess their performance. We selected and tested 3 apparently credible apps: the single available web-based app, one general reptile identification app, and one snakebite-specific identification app selected at random from the existing options. Apps with unrealistic claims or consistently poor user ratings were excluded, ensuring only reasonably credible applications were included. To cover a wide range of species from different regions, we used 2 images for each of the 12 most medically significant snake species (as defined by the WHO [18]), resulting in 24 test images. These species were (1) *Naja kaouthia*, (2) *Crotalus durissus*, (3) *Calloselasma rhodostoma*, (4) *Dendroaspis polylepis*, (5) *Daboia russelii*, (6) *Bitis arietans*, (7) *Echis ocellatus*, (8) *Naja haje*, (9) *Bothrops asper*, (10) *Pseudonaja textilis*, (11) *Oxyuranus scutellatus*, and (12) *Bungarus candidus*. We used verified photos from iNaturalist (with the appropriate use rights) that reflect what a typical user might upload [19]. Responses for each image were recorded as shown in Multimedia Appendix 5. We divided the results into 5 categories. The first category, “correctly identified,” was used when the species in the image was correctly named (eg, when an image of *Bitis arietans* was identified as *Bitis arietans*). The second category, “misidentified as a nonvenomous snake,” was used when a venomous species was incorrectly labeled as nonvenomous (eg, when *Bitis arietans* was misidentified as *Aspidites ramsayi*). The third category, “misidentified as another venomous snake,” included cases where the species was confused with another venomous species (eg, when *Bitis arietans* was identified as *Daboia russelii*). The fourth category, “misidentified as nonsnake,” was assigned when the image was classified as something other than a snake (eg, a worm). The fifth category, “NA,” was used when no response was generated due to technical issues.

The included applications were grouped according to their platform type (mobile- or web-based), regional focus (WHO regions), and features offered.

## Results

### Overview

**Figure 1.**
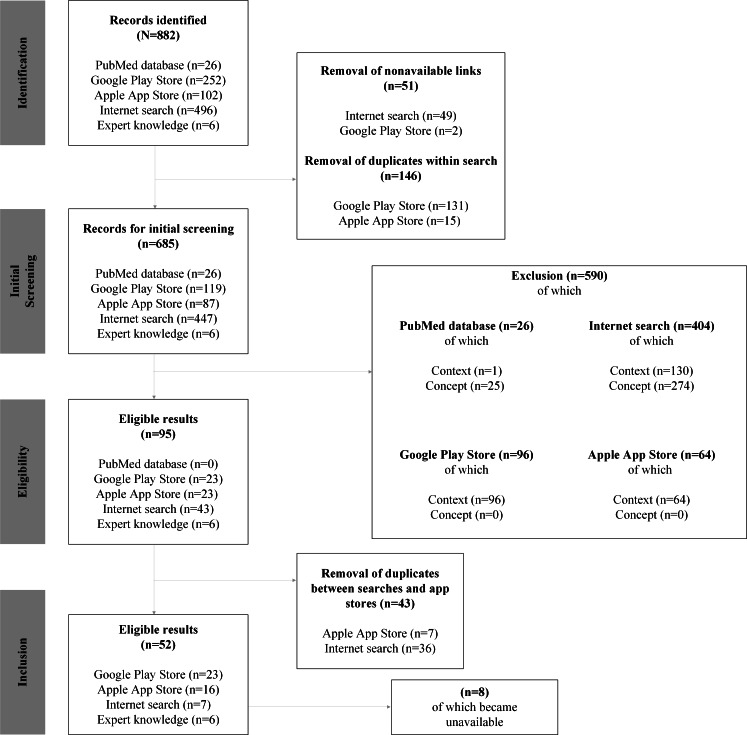
Flowchart of the digital health applications (DHAs) identified during screening. Reasons for exclusion were categorized as (1) context: not relevant for snakebite envenoming; (2) concept: not an interactive DHA; or (3) unavailable: not (or no longer) available (eg, broken link, 404 error, or security warning).

A total of 882 results were identified, including those from the PubMed database (n=26, 2.9%), Google Play Store (n=252, 28.6%), Apple Store (n=102, 11.6%), Google Search (n=496, 56.2%), and expert knowledge (n=6, 0.7%; Figure 1). After removing duplicates within single searches (n=146, 16.6%) and results with nonfunctioning links (n=51, 5.8%), 685 (77.7%) records were screened using the eligibility criteria. All PubMed database (n=26, 2.9%) results were excluded either because they were not SBE-related or did not describe a DHA (eg, general publications about snakebites). App store results were excluded for lacking snake or SBE-related content (n=160, 18.1%; eg, snake games), and internet search results were excluded for not being SBE-related or not being interactive DHAs (n=404, 45.8%; eg, news on snakebite victims). After removing cross-search duplicates (n=43, 4.9%), 52 (5.9%) results remained: 39 (39/52, 75%) from app stores (23/39, 59% Google Play Store and 16/39, 41% Apple App Store), 7 (13.5%) from the internet search, and 6 (11.5%) from expert knowledge (Figure 1). Of these, 94.2% (49/52) were mobile apps and 5.8% (3/52) were web-based platforms. Of the 49 mobile apps, the oldest was released in 2015, while 28 (57.1%) apps were released since 2019.

Of the 52 applications, 32 (61.5%) were snakebite-specific, while 20 (38.5%) were general applications that included snake-related content. Key features identified in these apps included snake identification (n=41, 78.8%), first aid advice (n=27, 51.9%), habitat information (n=18, 34.6%), prevention advice (n=8, 15.4%), and advanced treatment advice (n=7, 13.5%). A total of 18 (34.6%) applications included additional features, such as snake removal contacts or lists of hospitals with antivenom.

### Mobile Apps

Most mobile apps targeted a specific WHO region: South-East Asia (11/49, 22.4%), the Americas (9/49, 18.4%), Western Pacific (5/49, 10.2%), Africa (3/49, 6.1%), and Europe (1/49, 2.0%); 20 (40.8%) were nonregion specific or the region was unclear. We divided these 20 apps into two categories: (1) worldwide region, which includes apps with a clear global focus as indicated by the provided information or images, and (2) unclear region, which includes apps for which the regional scope could not be determined from the provided information or images. App details are summarized in Table 1.

**Table 1.** Description of mobile apps.

Region and app name	Prevention	First aid	Advanced treatment	Snake identification	Habitat	Other
**South-East Asia**						
Indian Snakes				✓	✓	
SARPA	✓	✓		✓		✓
SERPENT by Indiansnakes		✓	✓	✓	✓	✓
Snake Bite Awareness App		✓		✓	✓	✓
Snake bite prevention and rescue	✓	✓		✓		✓
Snake Bite Treatment						✓
Snake Helpline		✓		✓		✓
SnakeHub		✓		✓	✓	✓
Snakelens^a^		✓		✓^b^		
Snakepedia		✓	✓	✓	✓	✓
The Snakebite Assistant	✓	✓	✓	✓	✓	
**Americas**						
Asp Snake Identifier - USA	✓	✓		✓	✓	
GOES Health: Outdoor First Aid^c^		✓		✓		
Redtox App	✓			✓	✓	✓
SERPENT Brazil		✓		✓		✓
Snake Patrol Suriname				✓	✓	
SnakeBite911	✓	✓	✓	✓		
Snakes of North Carolina				✓	✓	
SnakeSnap!^c^				✓		
Suriname Snakebite Initiative^a^		✓	✓			
**Western Pacific**						
Australian Bites and Stings	✓	✓		✓		
Australian Snake ID^c^				✓	✓	
Field Guide to Victorian Fauna				✓		
First Aid		✓				
iFirstAid		✓				
**Africa**						
ASI Snakes		✓		✓	✓	✓
eSnakes Southern Africa^c^	✓	✓		✓	✓	
First Aid Africa		✓				
**Europe**						
Reptiles and Amphibians of Sweden		✓		✓	✓	
**Worldwide**						
Animal & Plant ID・EarthSnap^c^				✓^b^		
EveryScan: Identify Anything^c^				✓^b^		
iNaturalist				✓	✓	
Seek by iNaturalist				✓		✓
**Unclear**						
Army First Aid^c^		✓				
Automatic Snake Identifier				✓^b^		
Emergency Techniques & Guides^a^		✓				
First Aid and Emergency Techni		✓				
First Aid Guide Offline^c^		✓				
Frog Identifier Reptile ID^c^				✓^b^	✓	
Frst Aid | Emergency Med Aid^a^		✓				
Monosha^a^						✓
Picture Nature: Animal ID^c^				✓^b^	✓	
PIdentify^a^				✓^b^		
Snake ID - reptile identifier^c^				✓^b^		
Snake Identifier^c^				✓^b^		
Snake Identifier^a^				✓^b^	✓	
Snake Identifier: AI Scanner^c^				✓^b^		
Snake name - identify snakes^c^				✓		
Snake Species Identifier^a^				✓^b^		

a Not available anymore.

b Artificial intelligence–based photo identification.

c Cost involved.

### South-East Asia

Eleven apps were identified for South-East Asia (India n=10, 90.9% and Bangladesh n=1, 9.1%), and all were freely accessible. The app for Bangladesh included information on snake species, first aid advice, hospital and snake removal contacts, and educational content [20].

For India, 5 (50%) apps were designed for nationwide use, while 3 (30%) were specific to Kerala, 2 (20%) to Odisha, and 1 (10%) to West Bengal. The countrywide apps featured AI-assisted snake identification [21], field guides [22,23], and apps for snakebite management, including hospital and snake removal contacts [24,25]. The Kerala apps included a field guide with expert support and resource lists [26], first aid advice and emergency contacts [27], and a hospital search app with information on antivenom availability [28]. The apps for Odisha [29] and West Bengal [30] offered local snake information, snakebite symptoms, first aid advice, and snake removal contacts. The West Bengal app also listed hospitals with antivenom. Download numbers were available for all apps, ranging from 500 [28] to 100,000 [26].

### Americas

Nine apps were identified for the Americas (United States: n=5, Suriname: n=2, Mexico: n=1, and Brazil: n=1). Seven apps were free, and 2 for the United States had in-app purchases [31,32].

For the United States, 4 apps were designed for nationwide use, and 1 was specific to North Carolina. Three supported snake identification [31,33,34]: 2 field guides that also contained first aid advice [33,34], and 1 that claimed to offer AI-based snake identification with expert verification [31]. One app focused on prevention and first aid specifically for North American pit vipers [35], while the remaining app offered general first aid advice and information on snakebite prevention [32].

The apps for Mexico, Brazil, and Suriname provided first aid advice, expert contacts, and antivenom information. The Mexican app focused on venomous scorpions, spiders, and snakes, as well as expert contacts [36]. The Brazilian app offered SBE victims and medical personnel first aid advice and snake reporting [37]. One Suriname app offered first aid advice and treatment information for SBE victims and medical professionals [38], while the other was a field guide with first aid advice [39]. Download numbers were available for 4 of these apps, ranging from 10 [39] to 100,000 [31].

### Western Pacific

All 5 apps for the Western Pacific were specific to Australia, with 1 specific to the state of Victoria. Most were free, with 1 snakebite-specific app available for US $6.99 [40].

Three apps offered snake identification features: 2 field guides [40,41], and 1 with additional educational resources, first aid advice, and prevention [42]. Two others offered general first aid advice, including specific advice for snakebites [43,44]. Download numbers were available for 4 of these apps, ranging from 100 [40] to 50,000 [40,42].

### Africa

Three apps were identified for Africa: 2 were free [45,46], and 1 cost US $15.99 [47]. All offered first aid advice. Two apps were designed for Southern Africa (without defining specific countries). These included an interactive, comprehensive field guide [47] and a basic snake identification guide with expert identification and contacts for snake removal [45]. The third was a general first aid app for Africa, which included specific advice for snakebites [46]. Download numbers were available for 2 of these apps, ranging from 1000 [46] to 100,000 [45].

### Europe

One app was identified for Europe, specifically designed for Sweden. This free app serves as a field guide offering snake identification, habitat information, and basic first aid advice [48].

### Worldwide

All 4 apps identified for worldwide use offered snake identification features. Two were general AI-based identification apps with optional in-app purchases [49,50]; the other 2 were free and used crowdsourced identification, 1 included habitat information [51], and the other used gamification [52]. Download numbers were available for the 2 crowdsourced apps, ranging from 1 million [52] to 5 million [51].

### Unclear

Sixteen apps had an unclear regional focus. Most were free, while 6 (37.5%) apps involved a cost. A total of 10 (62.5%) offered snake identification features: 8 (80%) were snake-specific [53-60], and 2 (20%) were general animal apps [61,62]. Five (31.2%) were general first aid apps, which included specific advice for snakebites [63-67], and 1 (6.2%) collected snakebite data and linked snakebite victims to hospital care [68]. Download numbers were available for 6 of these apps, ranging from 100 [68] to 100,000 [65,66].

### Web-Based Platforms

Only 1 web-based platform was identified: “AI.Snakes,” which offered advanced treatment information, AI photo-based and clinical assessment–based identification, and regional risk assessments [69].

Two global platforms were added through author team expert knowledge [70,71], as they were not retrieved though the search strategy: the WHO’s “Snakebite Information and Data Platform” with information for up to 373 snake species, including distribution maps, antivenom, and clinical guidance; and the “VAPAGuide,” a free expert resource with emergency protocols, clinical flow charts, and species data. The web-based platforms and their functions are summarized in Table 2.

**Table 2.** Dedicated web-based platforms.

Region and platform name	Prevention	First aid	Advanced treatment	Snake identification	Habitat	Other
**Eastern Mediterranean**						
AI.Snakes			✓	✓^a^		
**Worldwide**						
Snakebite Information and Data Platform				✓		✓
VAPAGuide		✓	✓	✓		

a Artificial intelligence–based photo identification.

### Snake Identification

Of the 41 DHAs offering snake identification features, 17 (17/41, 41.5%) enabled photo uploads for identification, of which 2 (2/17, 11.8%) relied on expert identification [31,45], 2 (2/17, 11.8%) on identification by online communities [51,52], 13 (13/17, 76.5%) claimed to offer AI identification [21,49,50,53-59,61,62,69], 15 (15/41, 36.6%) displayed snake images or photos, 5 (12.2%) were field guides, and 1 (2.4%) used algorithms based on clinical symptoms and described snake morphology (eg, scale patterns) [24]. The identification process was not described in 3 apps (7.3%) [22,30,60].

For 4 AI-based DHAs, developer credentials and algorithm accuracy were unclear [21,54-56]. One claimed accuracy of more than 100% for each identification result [58], 4 apps were no longer available when we attempted to evaluate the feature [21,54-56], and some received user criticism in reviews based on the algorithm’s randomness in the results [53].

On the basis of our explorative assessments of 3 selected AI-based snake identification applications, the performance of this feature was highly variable. The “AI.Nature” application failed to return a result for any of the 24 photos used. The “Snake ID – reptile identifier” correctly identified 2 images but failed to detect a snake in 5 images, misidentified the snake as nonvenomous in 10 images, and suggested the wrong venomous species in 7 images. The “Frog Identifier Reptile ID” app correctly identified 13 images but failed to detect a snake in 1 image, misidentified the snake as nonvenomous in 1 image, and suggested the wrong venomous species in 9 images. Performance results and images used are shown in detail in Multimedia Appendix 5.

### First Aid

A total of 27 applications featured first aid advice for snakebites. These included 15 (55.6%) snake-specific apps, 9 (33.3%) general first aid apps with snakebite content, 2 (7.4%) general reptile guides, and 1 (3.7%) app on bites and stings. During the evaluation period, 5 (18.5%) apps were not accessible, and 2 (7.4%) were no longer available in the app store. Therefore, we were unable to evaluate them. This resulted in 20 (74.1%) apps being available for download and assessment. Of those, 3 (15%) covered all recommended first aid steps [23,24,26]. Most advised reassuring the bitten person, remaining calm, minimizing movement, and seeking medical care, but many omitted warnings about traditional or unsafe treatment (n=10, 50%) or the importance of moving away from the snake (n=12, 60%). Three (15%) apps incorrectly recommended washing the wound, and the “Army First App” advised killing the snake for identification and using a constricting band. Three (15%) apps provided minimal or no information [64,66,67]. Full details are provided in Multimedia Appendix 4.

## Discussion

### Principal Findings

This scoping review presents the first structured overview of DHAs for SBE and represents one of the earliest systematic efforts to characterize DHAs for NTDs with a breakdown of available features [13]. We identified 52 DHAs relevant to snakebite treatment or prevention, most of which were offered as snakebite-specific mobile apps. The most common features included snake identification and first aid advice.

Despite the number of apps available, their distribution does not align with the global burden of SBE. High-incidence regions, such as sub-Saharan Africa, remain underrepresented, with only 3 apps available for the region, while low-incidence countries, such as the United States and Australia have 5 apps each. This reflects the broader disparities in funding, research capacity, treatment infrastructure, and epidemiological data [72]. Most African countries lack reliable data on SBE, limiting effective policymaking and resource allocation [73]. This underrepresentation of the African continent extends to the nondigital space. Despite promising initiatives, snakebite management in Africa remains largely underfunded and underresourced [72]. Access to smartphones and reliable internet is increasing across the African continent, making DHAs increasingly useful, particularly for health care workers [74]. Nevertheless, access remains limited, especially in rural populations, which continues to restrict the utility of DHAs for those populations. This contrasts with better rural connectivity observed in South Asia and the Western Pacific [75]. However, beyond India and Australia, these regions still face limited availability of snakebite-related DHAs.

First aid and snake identification were the most common features. Most apps’ first aid features were aligned with established guidelines, and only one app included several contraindicated practices [63]. However, transparency around authorship, clinical validation, and accountability was often lacking, raising safety concerns. These apps could be improved by referencing international guidelines, linking sources, and warning against harmful practices. Because many people self-treat using unsafe methods, validated content could improve outcomes and support the WHO’s pillar to empower communities with better knowledge and applications.

Despite reported advances, the availability of AI-based snake identification apps remains limited and user-facing performance is variable. Challenges, such as interspecies similarities, species variation, and poor image quality, make accurate identification difficult. Nevertheless, research prototypes trained on large image datasets have reported high classification accuracy across many species and in real-world images [76-78]. However, even occasional misidentifications pose serious risks if used for medical decisions after snakebites. In our exploratory assessment, using verified images loaded into 3 apps which claimed to use AI for snake identification, outputs were inconsistent, with frequent misidentifications or no results (Multimedia Appendix 5), suggesting limited real-world usability of current AI-claimed snake identification apps. None of the reviewed apps offering AI-based snake identification disclosed details on development or evaluation details (eg, training data, validation datasets, and versioning), and most relied on internally derived confidence scores rather than externally validated outcomes. These gaps highlight the mismatch between research-level AI and published apps [79].

Few apps disclosed their development purpose or process, and many were developed outside health systems, lacked regulatory oversight, and included liability disclaimers, indicating the need for stronger regulation. Several apps also lacked emergency-friendly interfaces, relying on long, unstructured PDFs that pose risks during time-sensitive situations. Despite these limitations, apps with expert input and clear scope hold promise, especially if further developed within regulatory frameworks. Only 1 app was described in a peer-reviewed publication, highlighting the general lack of scientific documentation or transparency in app development [80]. This manuscript was published after our analysis and was therefore not identified through our initial search.

### Recommendations

The review identifies 4 priority actions to improve the quality, accessibility, and alignment of DHAs for SBE. Responsibility should be considered on multiple levels: national health authorities and professional societies can define minimum clinical content standards and integration pathways, app marketplaces can require baseline transparency fields as listing criteria, and global actors can support shared taxonomies and registries to improve discoverability and interoperability.

First, enforcing basic transparency and documentation standards, such as publishing development histories, data sources, and use context, would enable consistent assessment. Co-designing apps with target user groups, particularly underserved groups, would ensure relevance and usability. Second, a global digital health registry for SBE and other NTDs could enhance visibility, reduce duplication, support interoperability, and promote uptake. Third, incentivizing open collaboration (eg, co-design, open-source codebases, stakeholder involvement, or version control) would improve usability and trust. Fourth, aligning app development with national digital health strategies and systems would strengthen sustainability and institutional support.

### Strengths and Limitations

This study has several limitations. First, reliance on real-time data extraction rather than app downloads may have led to the extraction of incomplete data. However, this only applies to data from apps no longer accessible, illustrating the app marketplace’s volatility, particularly in niche or fast-growing areas. Second, the user-centric search strategy may have missed less visible resources. Notably, 2 expert platforms known to the author team were not identified in the search and were only included via expert knowledge [70,71]. The inability to find these platforms, despite using a very broad search strategy and searching in multiple languages, probably reflects challenges that typical users may also face. Visibility of apps and platforms is influenced by algorithmic ranking and varying levels of search engine optimization, and not by the quality of the DHA itself. End users and frontline health workers operating under time pressure and resource constraints are unlikely to find these platforms with a simple search and have even fewer cues to judge whether tools are trustworthy and fit for purpose, an especially acute challenge in neglected disease settings.

Furthermore, our search strategy may have overlooked digital tools without a direct web presence, such as chatbots running on platforms such as WhatsApp [81]. It also excluded other service-based digital solutions, such as remote consultation platforms, including the Remote Envenomation Consultancy Services in Malaysia [82]. Although this is a limitation, the lack of an online presence meant that this type of DHA could not be directly analyzed. Its content was not readily accessible and therefore falls outside the scope of this review, as this was an inclusion criterion. Finally, we did not download, evaluate, or compare all available apps, as currently no standardized framework exists for assessing snakebite apps, and any such comparison would have been inherently subjective. Despite these constraints, the gray-inclusive strategy and registered protocol support reproducibility. Findings align with WHO priorities on transparency, interoperability, and equity in digital health [5,83,84].

This review identified critical gaps in transparency, metadata completeness, standardized evaluation, efficacy metrics, and alignment of digital snakebite apps with health governance frameworks. Many apps lacked documented development processes, references to clinical standards, or integration with national health strategies. These deficits undermine long-term sustainability, impede user trust, and hinder integration into structured care pathways.

Geographic misalignment, particularly underrepresentation in high-burden regions such as sub-Saharan Africa, along with persistent digital inequities in low- and middle-income countries, further highlight the disconnection between needs and app availability. Without targeted efforts to strengthen DHA and address usability barriers in low- and middle-income countries, such apps risk reinforcing exclusion rather than improving access [7]. Infrastructure challenges, regulatory gaps, and poor interoperability further complicate integration into formal care systems.

Despite pervasive gaps in transparency, quality, and integration, the findings from this review provide a replicable foundation for future assessment and strategic planning of digital applications in the field of SBE. Scaling this framework across NTDs and embedding shared standards can support equitable, evidence-based digital innovation in neglected disease areas.

## Supplementary material

10.2196/83744 Multimedia Appendix 1 Search queries.

10.2196/83744 Multimedia Appendix 2 Data extraction sheet.

10.2196/83744 Multimedia Appendix 3 Adaptations from protocol.

10.2196/83744 Multimedia Appendix 4 First aid testing.

10.2196/83744 Multimedia Appendix 5 Artificial intelligence picture testing.

10.2196/83744 Checklist 1 PRISMA-ScR checklist.

## Abbreviations

AI: artificial intelligence
DHA: digital health application
DHI: digital health intervention
NTD: neglected tropical disease
PRISMA-ScR: Preferred Reporting Items for Systematic Reviews and Meta-Analyses Extension for Scoping Reviews
SBE: snakebite envenoming
WHO: World Health Organization
