# Supplementary material for: Digital Health Apps and Web-Based Platforms to Support the Prevention and Management of Snakebite Envenoming: Scoping Review
Source: JMIR Mhealth Uhealth. 2026 Jun 2;14:e83744. doi: 10.2196/83744 (PMC13229464; doi:10.2196/83744)
Supplement: Multimedia Appendix 1 [file mhealth-v14-e83744-s001.pdf]

Appendix 1 - Search queries

English (en)

Pubmed
(“snakebite” OR “snake bite”) AND (“digital health” OR “telemedicine” OR “telehealth” OR “mHealth” OR “m-Health” OR “eHealth” OR “E-Health” OR “health app” OR “digital health intervention” OR “digital health technology” OR “healthcare app” OR “mobile health app” OR “remote healthcare” OR “digital diagnosis” OR “digital treatment” OR “digital tool” OR “digital surveillance” OR “digital monitoring” OR “AI”)

App Stores
“snakebite”, “snake bite”, “snake identification”, “snake surveillance”, “snakebite surveillance”, “snakebite diagnosis”, “snakebite treatment”, “snakebite first aid”, “snakebite education”, “snakebite prevention”

Internet search
“app” OR “digital health” OR “eHealth” OR “mHealth” AND “snakebite” OR “snake bite”

Internet search in other languages:

French (fr)
“application” OR “santé numérique” OR “e-Santé” OR “mSanté” AND “morsure de serpent”

Spanish (es)
“aplicación” OR “salud digital” OR “eSalud” OR “mSalud” AND “mordedura de serpiente”

Arabic (ar)
“التطبيق” OR “الصحة الرقمية” OR “الصحة الإلكترونية” OR “م-الصحة” AND “لدغة الأفعى” OR “لدغة الثعبان”

Portuguese (pt)
“aplicativo” OR “saúde digital” OR “e-Saúde” OR “m-Saúde” AND “picada de cobra” OR “mordida de cobra”

Hindi (hi)
“ऐप” OR “डिजिटल स्वास्थ्य” OR “ई-हैल्थ” OR “एम-स्वास्थ्य” AND “साँप का डंक”

Vietnamese (vi)
“ứng dụng” OR “sức khoẻ kỹ thuật số” OR “y tế điện tử” OR “y tế di động” AND “rắn cắn”

Thai (th)
“แอปพลิเคชัน โปรแกรมประยุกต์ในระบบคอมพิวเตอร์” OR “สุขภาพดิจิทัล” OR “เทคโนโลยีสารสนเทศสุขภาพ” OR “สุขภาพเคลื่อนที การใช้เทคโนโลยีมือถือไร้สายเพื่อสุขภาพ” AND “งูกัด งูพิษกัด”

Indonesian (id)
“aplikasi” OR “kesehatan digital” OR “kesehatan elektronik” OR “mHealth” AND “gigitan ular”

Filipino (tl)
“app” OR “digital na kalusugan” OR “eKalusugan” OR “mKalusugan” AND “tuklaw ng ahas” OR “kagat ng ahas”

Korean (ko)
“애플리케이션” OR “디지털 건강” OR “인터넷을 통한 원격진료” OR “모바일 기기를 활용한 건강 관리” AND “뱀에 물린 상처” OR “사교상”

Afrikaans (af)
“toep” OR “digitale gesondheit” OR “eGesondheid” OR “mGesondheid” AND “slangbyt”

Swahili (sw)
“protu” OR “afya ya kidijitali” OR “eHealth” OR “mHealth” AND “kuumwa na nyoka”

Persian / Farsi (fa)
“تشخیص دیجیتال” OR “سلامت دیجیتال” OR “سلامت الکترونیک” AND “مارگزیدگی” OR “نیش مار”
