# Supplementary material for: Digital Health Apps and Web-Based Platforms to Support the Prevention and Management of Snakebite Envenoming: Scoping Review
Source: JMIR Mhealth Uhealth. 2026 Jun 2;14:e83744. doi: 10.2196/83744 (PMC13229464; doi:10.2196/83744)
Supplement: Multimedia Appendix 3 [file mhealth-v14-e83744-s003.pdf]

Appendix 3 – Adaptions from protocol

During the search and data extraction process, we decided to make some adaptions to our protocol where necessary or considered beneficial. These changes and reasoning are outlined here.
Additions to data extraction in the protocol:

- A text field "Comments" was added to allow for descriptive comments, where considered helpful.
- A data field "PCC Exclusion" with the options "Population, Concept, Context, NA", was added to facilitate a straightforward overview of the primary reason for exclusion according to the PCC concept. The selection of "NA" (Not Applicable) was added by us to denote search results that were blocked, unavailable, flagged as a security risk, paywalled, or were otherwise not accessible. A text field "Specify exclusion reason" was added to facilitate a straightforward reasoning for exclusion in addition to "PCC Exclusion" (e.g., game, other app).
- If the internet search returned a result for an app that was not previously found via the appstore searches, we assumed a restriction (e.g., geoblock) and searched for a link to an appstore by searching for the name of the app on the internet to include the result.
- A text field "Page Author (Web and PubMed)" was added, to avoid confusion in cases where the information was conflicting with the field "Publisher" for apps.
- A text field "Page Title" was added to avoid confusion in cases where the information was conflicting with the field "App Title".
- The text field "Link" was split into "Link (tool)" and "Link (search)" to allow the recording of different links leading to the same tool and quick access to the used weblink for potential later evaluations.
- A text field "Country focus" was added to record more precise location information beyond the scope of the field "Spatial focus", where such information was available.
- A text field "Site type" was added for internet results flagged for inclusion, to record the basic type of website (e.g., news, shop, etc.), where such information could be reasonably derived.
- A field "Site update" was added for internet results flagged for inclusion, to record the year of publication or last update, where such information was available.
- A data field "Snakebite specific/focused tool" with the options "Yes, No" was added to indicate whether a tool was considered snakebite management specific or merely referred to snakebite in a broader context (e.g., general first aid or ID tools, which mentioned snakes to some degree).
- For found appstore results, several fields were added to record additional "low hanging fruit" information that was potentially available but not considered important to our study when developing the protocol. The added fields for Google Play Store (AppGPS) are "Rating (x of 5), Ratings count (range), Downloads (range), Store category, Version Mobile, Version Computer,

Android Version Minimum, Release date, Last update mobile, Last update computer". The added fields for Apple App Store (AppApple) are "Rating (x of 5 stars), # of ratings, Downloads (range), Store category, Version Mobile, Version Computer, iOS Version Minimum, Release date, Last update mobile, Last update computer". To allow for a comparable snapshot of such data at the same time, this information was collected on the same day (November 18, 2024), once the primary data extraction was completed.

Omissions from data extraction in the protocol:

- The data field "Availability" was omitted, due to the impracticability of reliably determining if and why non-returned results were blocked (by e.g., language, location, device or due to other reasons). This was cross-checked with tools known to exist but not returned by the search. Before the omission, we attempted to circumvent the access barriers by trying different VPN services on different mobile and desktop devices, but the feasibility was defeated by unreliable and non-reproducible results.
- The data field "Languages (multiple)" was omitted due to the impracticality of determining if multiple language results were automatically translated or if the content was written or reliably translated in the presented languages.
- The data field "User interaction" was omitted, since the level of user interaction could not be properly assessed, as such information could not be reliably extracted from the descriptions given. Further evaluation was considered, but would have required the individual installation and systematic assessment of found tools/applications, which was beyond the scope of this review.

Between the finalisation of the protocol and execution, changes from the service providers required adaptations to the protocol to be executed properly. These adaptations are listed here:

- Google changed the default display of search results from an 'infinitely scrolling' page to individual pages with 10 results each. As an adaptation, we saved the search results pages individually to achieve the intended 50 results (where sufficient results were returned).
- The Apple App Store could no longer be searched independently of the login (i.e., it required logging in to search). To mitigate this, we used a different search provider (fnd.io) to search this app store and follow the provided direct links to the Apple App Store for the evaluation.
