# Supplementary material for: Digital Health Apps and Web-Based Platforms to Support the Prevention and Management of Snakebite Envenoming: Scoping Review
Source: JMIR Mhealth Uhealth. 2026 Jun 2;14:e83744. doi: 10.2196/83744 (PMC13229464; doi:10.2196/83744)
Supplement: Multimedia Appendix 4 [file mhealth-v14-e83744-s004.pdf]

Appendix 4 – First aid testing

	Move slowly away from the snake	Stay calm and reassure the bitten person	Remove all tight items around the affected area	Reduce movement of the affected area	Leave the wound area (or bite mark) alone	Rush to the nearest health facility for medical treatment	DON’T tie the affected area to stop blood circulation	DON’T use traditional methods or any unsafe treatments
FirstAid Africa	✓	✓	✓	✓	-	✓	-	-
iFirstAid	-	✓	✓	✓	✓	✓	✓	✓
GOES Outdoor First Aid	✓	✓	✓	✓	-	✓	✓	✓
Army First Aid	X	✓	✓	✓	X	✓	X	(✓)
Frst Aid | Emergency Med Aid*	-	-	-	-	-	✓	-	-
First Aid	-	✓	-	✓	(✓)	✓	✓	-
First Aid and Emergency Techni	-	✓	✓	✓	X	✓	✓	✓
First Aid Guide Offline	-	-	-	-	-	✓	-	-
Emergency Techniques & Guides*	-	-	-	-	-	-	-	-
SnakeBite911	✓	-	✓	✓	✓	✓	✓	✓
eSnakes Southern Africa	-	✓	✓	✓	(✓)	(✓)	(✓)	-
Vapa Guide	-	✓	-	✓	-	-	-	-
The Snakebite Assistant	✓	✓	✓	✓	✓	✓	✓	✓
ASI Snakes	-	✓	-	✓	✓	✓	✓	-
Asp Snake Identifier - USA	✓	✓	✓	✓	X	✓	-	-
Snake Helpline	-	✓	✓	✓	✓	✓	✓	✓
SnakeHub	✓	✓	✓	✓	✓	✓	✓	✓
SERPENT by Indiansnakes	-	-	-	✓	✓	✓	✓	✓
Snakepedia	✓	✓	✓	✓	✓	✓	✓	✓
Australian Bites and Stings	✓	✓	-	✓	✓	✓	✓	-
SERPENT Brazil	NA	NA	NA	NA	NA	NA	NA	NA
Snake Bite Awareness App	NA	NA	NA	NA	NA	NA	NA	NA
Reptiles and Amphibians of Sweden	NA	NA	NA	NA	NA	NA	NA	NA
Snakelens*	NA	NA	NA	NA	NA	NA	NA	NA
Suriname Snakebite Initiative*	NA	NA	NA	NA	NA	NA	NA	NA
Snake bite prevention and rescue	NA	NA	NA	NA	NA	NA	NA	NA
SARPA	NA	NA	NA	NA	NA	NA	NA	NA

✓ Correctly included
(✓) Partly included
X Contra-indicated
\- Not mentioned
NA Not applicable (app could not be accessed)
* Not available
