# Supplementary material for: Digital Health Apps and Web-Based Platforms to Support the Prevention and Management of Snakebite Envenoming: Scoping Review
Source: JMIR Mhealth Uhealth. 2026 Jun 2;14:e83744. doi: 10.2196/83744 (PMC13229464; doi:10.2196/83744)
Supplement: Multimedia Appendix 5 [file mhealth-v14-e83744-s005.pdf]

Appendix 5 – AI Identification Test

	**AI.Nature**	**Snake ID - reptile identifier**	**Frog Identifier Reptile ID**
Photo 1 – Naja kaouthia<br>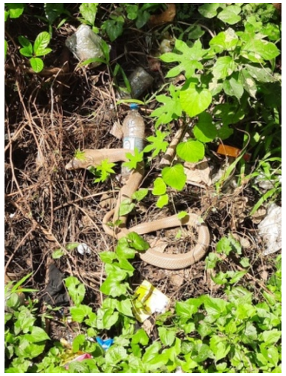<br>**Naja kaouthia © Nilutpal Das**<br>Source:<br>https://www.inaturalist.org/observations/273580259<br>Location: Mizoram, India	NA<br>(not loading)	X (wrong, no snake)<br>“This isn’t actually a snake at all but a legless lizard commonly called the “slow worm” (Anguis fragilis).”	X (wrong, venomous)<br>Accuracy: 9,55%<br>Ophiophagus hannah
Photo 2 – Naja kaouthia<br>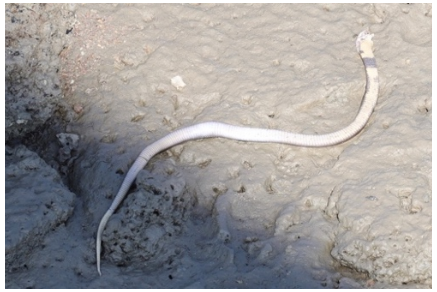<br>**Naja kaouthia by fishhead**<br>Source:<br>https://www.inaturalist.org/observations/198135057<br>Location: Kantrang, Thailand	NA<br>(not loading)	X (wrong, no snake)<br>“This is not a snake or any other reptile.”	X (wrong, venomous)<br>Accuracy: 6,21%<br>Clelia Clelia

Photo 3 – Crotalus durissus<br>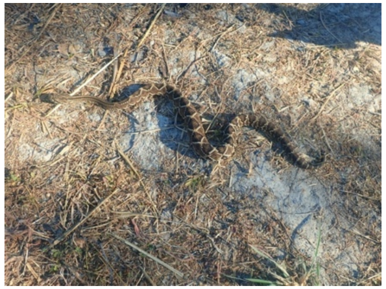<br>**Crotalus durissus by Tsssss**<br>Source:<br>https://www.inaturalist.org/observations/268847755<br>Location: Tocantins, Brasil	NA<br>(not loading)	X (wrong, non-venomous)<br>"This is a gopher snake (Pituophis catenifer), a non-venomous constrictor commonly found in dry, open country and edges of woodland."	✓<br>Accuracy: 25,46%<br>Crotalus durissus
Photo 4 – Crotalus durissus<br>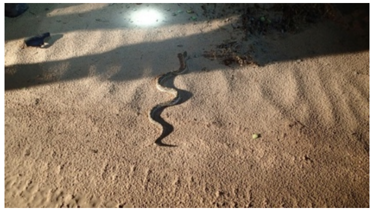<br>**Crotalus durissus by Allan Harris**<br>Source:<br>https://www.inaturalist.org/observations/110399393<br>Location: Zona Gral, Paraguay	NA<br>(not loading)	X (wrong, venomous)<br>"This is a venomous viper, almost certainly one of the small "sand" or "saw-scaled" vipers (genus Echis) rather than a harmless colubrid."	✓<br>Accuracy: 23,02%<br>Crotalus durissus
Photo 5 – Calloselasma rhodostoma	NA<br>(not loading)	X (wrong, non-venomous)<br>"This is a snake: it most closely matches the "brown-house snake" complex commonly kept around buildings in sub-Saharan Africa (genus Boaedon, formerly Lamprophis)."	✓<br>Accuracy: 95,52%<br>Calloselasma rhodostoma

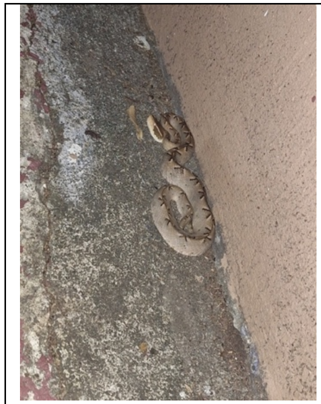<br>**Calloselasma rhodostoma © mihailovujic**<br>Source:<br>https://www.inaturalist.org/observations/263382211<br>Location: Krabi, Thailand			
Photo 6 – Calloselasma rhodostoma<br>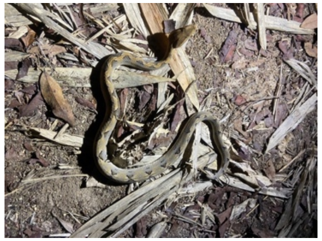<br>**Calloselasma rhodostoma © Phan Vũ Phúc Lân**<br>Source:<br>https://www.inaturalist.org/observations/223331166<br>Location: Vietnam	NA<br>(not loading)	X (wrong, non-venomous)<br>"This is definitely a snake—in fact it closely matches the look of a non-venomous "copperhead rat snake" (also called the Oriental ratsnake, scientific name Coelognathus radiatus)."	✓<br>Accuracy: 59,01%<br>Calloselasma rhodostoma
Photo 7 – Dendroaspis polylepis	NA<br>(not loading)	X (wrong, venomous)<br>"What you're looking at is almost certainly Pseudonaja textilis, commonly called the eastern brown snake."	✓<br>Accuracy: 37,38%<br>Dendroaspis polylepis

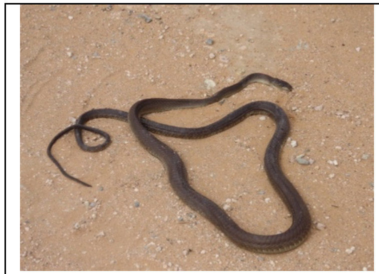 **Dendroaspis polylepis by dune_ninja** Source: https://www.inaturalist.org/observations/203760313 Location: Khomas Region, Namibia			
Photo 8 – Dendroaspis polylepis 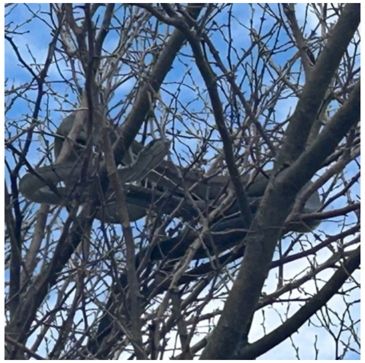 **Dendroaspis polylepis by Riana Fourie** Source: https://www.inaturalist.org/observations/255869742 Location: North-West, Botswana	NA (not loading)	X (wrong, no snake) "This isn't a snake or any kind of reptile—it looks like lengths of tubing or hose draped and tangled through the branches of that tree."	✓ Accuracy: 7,88% Dendroaspis polylepis
Photo 9 – Daboia russelii	NA (not loading)	X (wrong, non-venomous) "This is a juvenile ball python (Python regius), a non-venomous constrictor native to West and Central Africa but very commonly kept as a pet worldwide."	✓ Accuracy: 91,7% Daboia russelii

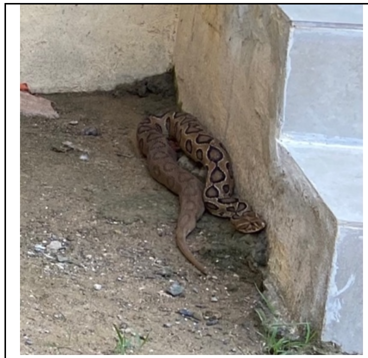<br>**Daboia russelii © Shivam Bhatt**<br>Source:<br>https://www.inaturalist.org/observations/180388798<br>Location: Gujarat, India			
Photo 10 – Daboia russelii<br>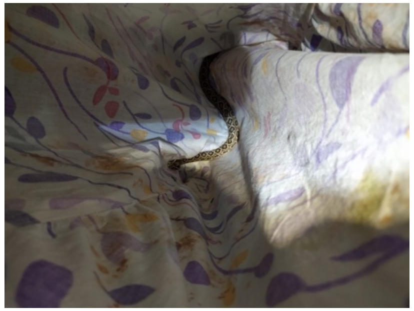<br>**Daboia russelii by Vijay Karthick**<br>Source:<br>https://www.inaturalist.org/observations/55182189<br>Location: Bangalore, India	NA<br>(not loading)	X (wrong, non-venomous)<br>This is in fact a small live snake—not a printed pattern or a toy. Based on the light-tan background color, the darker brown to black-edged oval “blotches,” relatively slender body and head proportions, it most closely matches a young Ball Python (Python regius), sometimes called the Royal Python.	✓<br>Accuracy: 24,94%<br>Daboia russelii
Photo 11 – Bitis arietans	NA<br>(not loading)	X (wrong, venomous)<br>“This is a venomous viper, not just an innocuous snake. In fact it closely matches the pattern and build of the Russell’s Viper (Daboia russelii).”	X (wrong, venomous)<br>Accuracy: 51,79%<br>Bothrops diporus

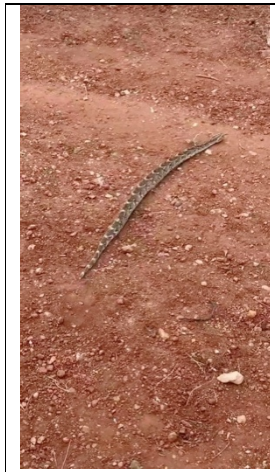<br>**Bitis arietans by Prince Molokomme**<br>Source:<br>https://www.inaturalist.org/observations/254961401<br>Location: Vhembe, South Africa			
Photo 12 – Bitis arietans<br>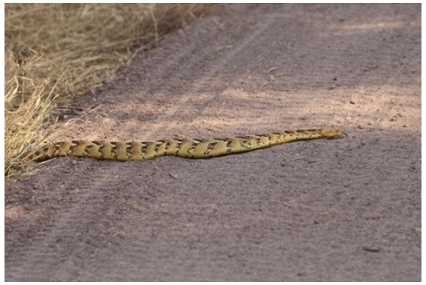<br>**Bitis arietans by Henggang Cui**<br>Source:<br>https://www.inaturalist.org/observations/179608246<br>Location: Lake Manyara, Tanzania	NA<br>(not loading)	X (wrong, non-venomous)<br>“This is a Woma python (Aspidites ramsayi), a medium-sized Australian python. Key features that give it away are the pale yellow-olive ground color with irregular, saddle-shaped brown blotches along the back, a gently tapering head that lacks the heat-sensing pits you’d see in many other pythons, and an overall length (in your photo) that appears to be well over a metre.”	✓<br>Accuracy: 25,39%<br>Bitis arietans
Photo 13 – Echis ocellatus	NA<br>(not loading)	X (wrong, venomous)<br>“This is not a harmless colubrid or “grass snake” at all but a small viper—and almost certainly one of the Asiatic pit-vipers (family Viperidae, subfamily Crotalinae).”	X (wrong, venomous)<br>Accuracy: 91,2%<br>Echis carinatus

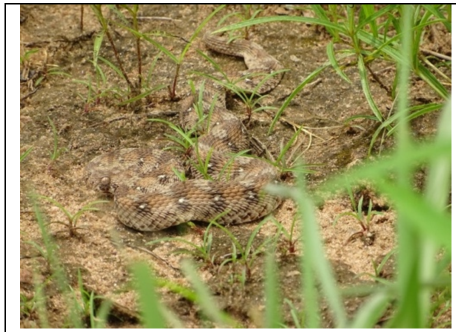<br>**Echis ocellatus © Mohamed SAOUPKAI**<br>Source:<br>https://www.inaturalist.org/observations/90591168<br>Location: Copargo, Bénin			
Photo 14 – Echis ocellatus<br>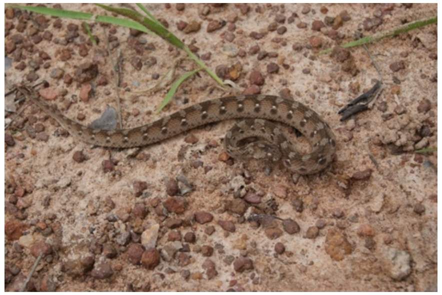<br>**Echis ocellatus © Wolfram Remmers**<br>Source:<br>https://www.inaturalist.org/observations/193609181<br>Location: Nahouri, Burkina Faso	NA<br>(not loading)	X (wrong, non-venomous)<br>“This is a young kingsnake, almost certainly a Prairie Kingsnake (Lampropeltis calligaster).”	X (wrong, venomous)<br>Accuracy: 87,95%<br>Echis carinatus
Photo 15 – Naja haje	NA<br>(not loading)	X (wrong, venomous)<br>“This is a recognizably colubrid snake coiled at the mouth of a rocky burrow.”	✓<br>Accuracy: 43,31%<br>Naja haje

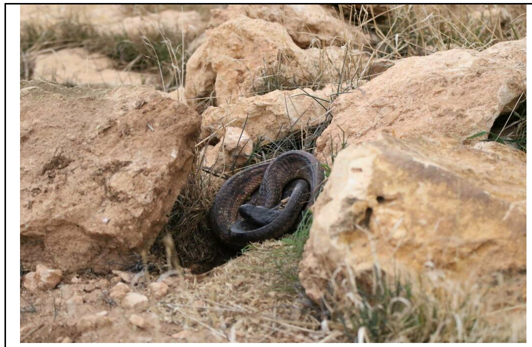<br>**Naja haje © Mourad Harzallah**<br>Source:<br>https://www.inaturalist.org/observations/152826952<br>Location: El Ma Labiodh, Algérie			
Photo 16 – Naja haje<br>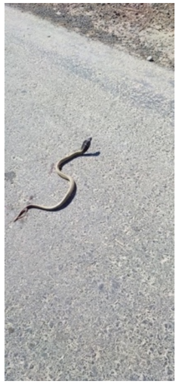<br>**Naja haje © Abdenour Kheloufi**<br>Source:<br>https://www.inaturalist.org/observations/99798490<br>Location: Sidi Okba, Algérie	NA<br>(not loading)	X (wrong, non-venomous)<br>“This image shows a slim, fast-moving whip snake rather than a viper or other venomous reptile. Based on the uniform olive-green to brownish dorsal coloring, the slender “whip-like” body and the characteristic way it flattens its neck when alarmed, it most closely matches what field herpetologists call the “green whip snake” (in Europe often Hierophis viridiflavus) or a closely related whipsnake (Dolichophis/Caspian whipsnake) depending on exact locale.”	X (wrong, non-venomous)<br>Accuracy: 65,42%<br>Masticophis fuliginosus
Photo 17 – Bothrops asper	NA<br>(not loading)	✓<br>“This is definitely a snake, and more precisely it appears to be a pit-viper of the Bothrops/“fer-de-lance” complex (often called the terciopelo in Central America).”	✓<br>Accuracy: 57,75%<br>Bothrops asper

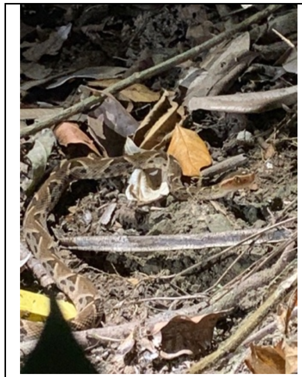<br>**Bothrops asper by Scott Loarie**<br>Source:<br>https://www.inaturalist.org/observations/20778192<br>Location: Panama, Panama			
Photo 18 – Bothrops asper<br>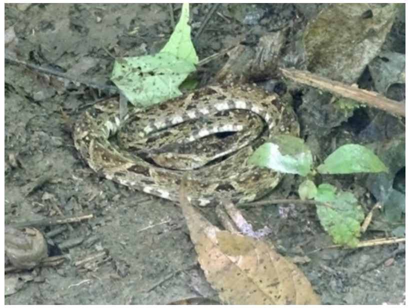<br>**Bothrops asper by Dario Taraborelli**<br>Source:<br>https://www.inaturalist.org/observations/36890016<br>Location: Colón, Panama	NA<br>(not loading)	X (wrong, venomous)<br>“This is a timber rattlesnake (Crotalus horridus).”	X (wrong, venomous)<br>Accuracy: 39,63%<br>Bothrops leucurus
Photo 19 – Pseudonaja textilis	NA<br>(not loading)	✓<br>“This little fellow is a juvenile eastern brown snake (Pseudonaja textilis), one of Australia’s most dangerous snakes.”	X (wrong, venomous)<br>Accuracy: 9,31%<br>Austrelaps superbus

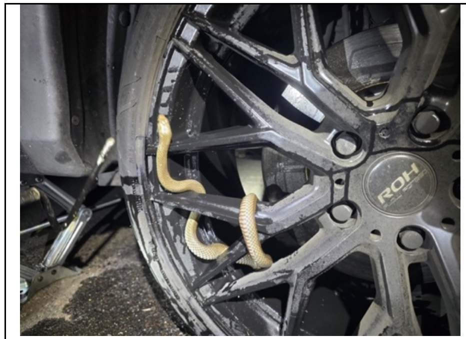<br>**Pseudonaja textilis by Max Tibby**<br>Source:<br>https://www.inaturalist.org/observations/258705699<br>Location: Mitcham, Australia			
Photo 20 – Pseudonaja textilis<br>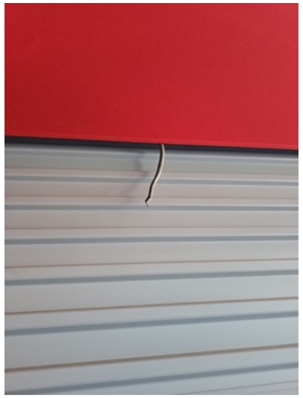<br>**Pseudonaja textilis by Max Tibby**<br>Source:<br>https://www.inaturalist.org/observations/257418280<br>Location: Mitcham, Australia	NA<br>(not loading)	X (wrong, no snake)<br>"This isn't a snake or any kind of reptile – it looks like a pull-cord (or similar cable) hanging down, not an animal."	X (wrong, no snake)<br>Accuracy: 21,75%<br>Boettgerilla pallens (worm slug)
Photo 21 – Oxyuranus scutellatus	NA<br>(not loading)	X (wrong, venomous)<br>"This is a brown-coloured elapid commonly known as an eastern brown snake (Pseudonaja textilis)."	X (wrong, venomous)<br>Accuracy: 27,67%<br>Pseudonaja mengdeni

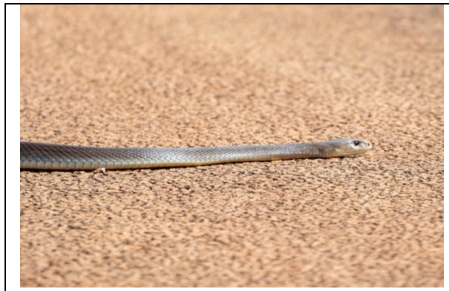 **Oxyuranus scutellatus by 松井 淳** Source: https://www.inaturalist.org/observations/255071530 Location: Cook, Australia			
Photo 22 – Oxyuranus scutellatus 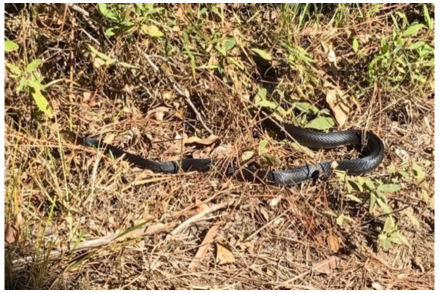 **Oxyuranus scutellatus © Jesse de Vries** Source: https://www.inaturalist.org/observations/175706427 Location: Livingstone, Australia	NA (not loading)	X (wrong, non-venomous) “This is indeed a real snake. Based on its slender, uniformly glossy black body, smooth scales, narrow tapering head and rapid “S”-shaped posture, it most closely matches the Eastern Black Racer (Coluber constrictor)—a common non-venomous colubrid in the southeastern United States.”	X (wrong, venomous) Accuracy: 35,22% Pseudechis porphyriacus
Photo 23 – Bungarus candidus	NA (not loading)	X (wrong, no snake) “This isn’t a snake or any other reptile.”	✓ Accuracy: 48,81% Bungarus candidus

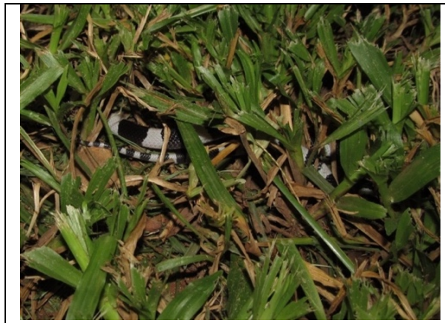<br>**Bungarus candidus © Derek Hennen**<br>Source:<br>https://www.inaturalist.org/observations/59103440<br>Location: Krabi, Thailand			
Photo 24 – Bungarus candidus<br>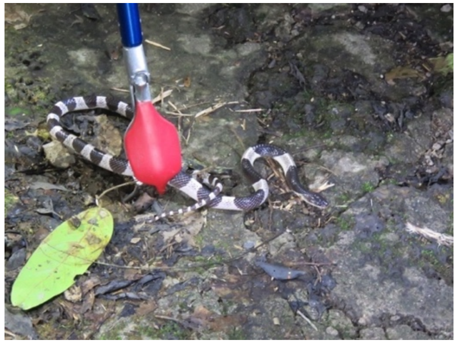<br>**Bungarus candidus © Audrey Kauppila**<br>Source:<br>https://www.inaturalist.org/observations/143232594<br>Location: Vietnam	NA<br>(not loading)	X (wrong, non-venomous)<br>"This is a banded kingsnake (genus Lampropeltis), most likely the Eastern (Lampropeltis getula) or California kingsnake (Lampropeltis californiae) complex – non-venomous, smooth-scaled constrictors recognizable by their alternating dark and light rings."	✓<br>Accuracy: 96,69%<br>Bungarus candidus
✓ Correctly identified<br>X Not correctly identified<br>NA Not identified			
